# Experiences and training needs of healthcare providers involved in the care of Ghanaian adolescents living with HIV: an interventional study

**DOI:** 10.1186/s12887-020-02086-w

**Published:** 2020-06-04

**Authors:** Anna Hayfron-Benjamin, Dorcas Obiri-Yeboah, Yemah Mariama Bockarie, Ernestina Asiedua, Ibrahim Baidoo, Angela D. Akorsu, Stephen Ayisi-Addo

**Affiliations:** 1 Department of Maternal and Child Health, School of Nursing and Midwifery, University of Cape Coast, Cape Coast, Ghana; 2 Department of Microbiology and Immunology, School of Medical Sciences, University of Cape Coast, Cape Coast, Ghana; 3 Department of Paediatrics, Cape Coast Teaching Hospital, Cape Coast, Ghana; 4 Department of Maternal and Child Health, School of Nursing, University of Ghana, Legon, Ghana; 5 The Public Health Unit, Cape Coast Teaching Hospital, Cape Coast, Ghana; 6 School for Development Studies, University of Cape Coast, Cape Coast, Ghana; 7 National AIDS/STI Control Program of the Ghana Health Service, Accra, Ghana

**Keywords:** Adolescents, ALHIV, Healthcare providers, Training needs, Ghana

## Abstract

**Background:**

Caring for adolescents living with HIV/AIDS (ALHIV) can be overwhelming due to their unique needs. Ghana is currently among nine countries in West and Central Africa contributing to 90% of new paediatric infections in the sub-region with a growing population of ALHIV. Regardless, gaps in paediatric related care including healthcare providers (HCPs) capacity issues have been identified. This study sought to assess the competencies of adolescent-oriented healthcare providers before, and after interventionist training to inform recommendation that would guide the psychosocial care they give to ALHIV.

**Methods:**

The study adopted a mixed methods approach with a non-randomized interventional study involving three-phase multi-methods. The sample consisted of 28 adolescent-oriented and multi-disciplinary healthcare providers at the Cape Coast Teaching Hospital (CCTH) in Ghana. Data were obtained in three phases, namely, a baseline survey, interventionist training, and post-training in-depth interviews. Quantitative data were analyzed using Stata version 13 for descriptive analysis while the qualitative data were analyzed thematically using NVivo version 11.

**Results:**

Although the majority of the HCPs claim to be knowledgeable about adolescent health issues (*n* = 21, 75.0%), only about a third (*n* = 10, 35.7%) could correctly define who an adolescent is. The majority (*n* = 18, 64.3%) had not received any training on how to work with the adolescent client. The main areas identified for improvement in the ALHIV care in phase 1 included issues with psychosocial assessment, communication and treatment adherence strategies, creating an adolescent-friendly work environment, and availability of job aids/protocols. During the post-training interviews, participants reported an improved understanding of the characteristics of an adolescent-friendly site and basic principles for ALHIV care. They were also able to correctly describe the widely used adolescent health assessment tool; the HEEADSSS. Post intervention interviews also revealed HCPs perception on increased practice related confidence levels and readiness to implement new knowledge and skills gained.

**Conclusion:**

This study has shown that targeted training on routine ALHIV care is effective in increasing HCPs knowledge, skills and confidence. Addressing the healthcare system/facility related gaps serves as an impetus for improved ALHIV care among HCPs.

## Background

Increased access to antiretroviral therapy (ART) has contributed to a significant increase in the number ALHIV world-wide with the majority from the Sub-Saharan African (SSA) region, where the disease burden exists [1–3]. However, due to the unique developmental challenges associated with the adolescence stage and long-term treatment specific issues, ALHIV face numerous unique and complex challenges [1–3]. The optimal management of HIV disease becomes complicated for many adolescents and their providers, as many ALHIV experience denial about their HIV disease, sometimes as a coping mechanism [2–4]. The incidence of non-compliance to a treatment regimen including ART is also increasing due to inadequate capacity to keep pace with the unique needs of this population [2–4]. These challenges may significantly impact on the pattern and process of change exhibited by these individuals as they grow through their formative years. As such, once HIV-positive adolescents are actively receiving medical care, the question of how best to treat them must be addressed [2–4]. This is necessary to prevent morbidity and mortality among ALHIV, arising from behavioral and psychological causes, which in some cases is worsened by unfavorable national policy and failures of health service delivery systems (including poor capacity building for relevant HCPs and limited or unavailability of relevant logistics or infrastructure to facilitate care provision) [2–4].

Identifying and addressing such barriers is important to ensuring a better care outcome for ALHIV, and by offerring adolescent-sensitive services, HCPs can make a significant diffeence [2–4]. These can be achieved through well-established ALHIV programs at clinical care centers. However, one of the gaps in the delivery of paediatric/adolescent HIV care services is the limited number HCPs who have adequate knowledge and skills to comfortably provide developmentally appropriate HIV counseling and related services including disclosure, provision of on-going supportive counseling, and addressing care and treatment adherence issues [5, 6]. The situation is particularly so in SSA, Ghana inclusive, where the number of HIV counselors trained in paediatric aspects of HIV counseling is limited. As such, most HIV care centers providing care to children/adolescents do so without providing the essential counseling support necessary to ensure good treatment outcomes [5–7].

In addressing the complexity associated with ALHIV, the WHO advocates for specific competency-based training for all HCPs involved with ALHIV care to enable them to provide high standard counseling and support services to them and their families [6–8]. Understanding of the HCPs challenges is an entry point to the review of existing programs, policies and working protocols as well as the adaption of evidenced-based best practices for quality improvement [1, 6, 9, 10]. Regardless, not much is documented about how well HCPs are prepared to provide services that meet the needs of ALHIV. Although a multitude of programs is currently focusing on scaling up paediatric HIV prevention, care, treatment, and support services, there is, however, urgent need to focus efforts on specific training for health workers to build the much-needed motivation, confidence, knowledge and skills to help manage such population [6, 7].

Ghana is currently among nine countries in West and Central Africa contributing to 90% of new paediatric infections in the sub-region and with a growing population of ALHIV as 40% of new HIV infections occur in this age group [4]. As at the end of 2017 about 28,000 children 0–14 and 19,000 adolescents 10–19 years were living with HIV in Ghana [11﻿, 1﻿2]. Regardless, gaps in paediatric related care including HCPs capacity training issues have been identified, and currently, a national paediatric task force team for a paediatric accelerated plan has been instituted to address such issues [4]. Like many other countries in the SSA region, there is also scarcely reported literature on how equipped HCPs are in addressing these unique psychosocial complexities of ALHIV. Hence the need to explore the HCPs knowledge and experiences with regards to ALHIV Care and to determine their training needs (such as on ALHIV specific knowledge, skills and care strategies) needed to improve the quality and standard of ALHIV care. This study therefore, sought to assess the competencies of adolescent-oriented healthcare providers before, and after interventionist training to inform recommendation that would guide the psychosocial care they give to ALHIV. The study will also generate the needed evidence that can inform programming for improved care for ALHIV, where gaps are identified.

## Methods

**Fig. 1:**
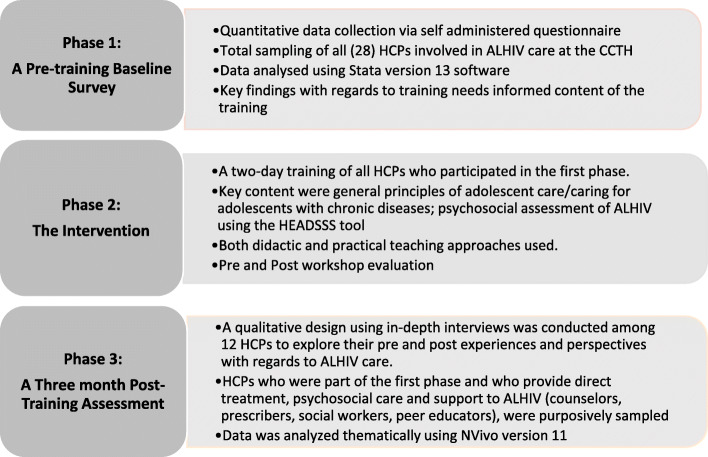
Schematic Diagram of the Study Project

A non-randomized interventional study involving three-phase multi-methods was conducted. Each phase was planned to address an objective of the study and the result of the research triangulated to form a comprehensive whole. Data were collected between July and December 2017. The three phases are schematically presented in Fig. 1.

### Phase 1: pre-training baseline survey

The objective of this first phase was to conduct a training needs assessment of the HCPs involved in ALHIV care. A survey was conducted among 28 HCPs of varied background using a pre-tested questionnaire to evaluate HCPs’ knowledge, attitudes, and experiences with regards ALHIV care. The tool specifically collected information on HCPs knowledge and practices with regards to the principles of adolescent-friendly services, the characteristics of the adolescence developmental stage and associated challenges, as well as strategies of effective communication, psychosocial assessment, and care. HCPs attitude towards care provision to the ALHIV client as well as their challenges with the care were also assessed.

All categories of HCPs rendering ART and other services to ALHIV assessing care at the Cape Coast Teaching Hospital were sampled and included in the study. These included mainly nurse/midwife prescribers and counselors, medical officers, medical social workers, biomedical scientists, pharmacists, and peer educators. With their expressed written consent all these HCPs were included in the study. This was a relatively smaller population; hence a census was used involving all members of the team working with adolescents at the unit during the study period.

Stata version 13 software (Stata Corp, Texas USA) was used to generate descriptive statistics for the socio-demographic characteristics and the gaps.

### Phase 2: the intervention

Findings from phase one informed a 2-days training, which targeted all categories of HCPs involved in ALHIV care at the CCTH. This capacity-building training emphasized the basic principles, policies, approaches, and strategies required for adolescent care, how to communicate effectively with adolescents with special needs and psychosocial assessment for ALHIV. Facilitative teaching via presentations, discussions, feedback questioning, participatory and transformative methods (such as case studies, role plays, and video shows) were employed. The facilitators were national level trainers for adolescent care with years of experience in conducting such trainings. They just engaged the participants in such a way to ensure that the desired outcome will be achieved [8].

### Phase 3: post training evaluation

Three months’ post-training on ALHIV care, an in-depth interview was conducted among a section of the participants who were part of the first phase and who provided direct treatment and psychosocial care to the ALHIV. The focus of the qualitative design was to explore their pre and post experiences and perspectives with regards to ALHIV psychosocial assessment and care. Knowledge and practice gaps identified in phase one informed the questions participants were asked during the interview.

The third phase comprised of 12 participants (mainly counselors including social workers, prescribers and peer educators), who were part of the first phase and who were purposively selected due to direct involvement in the treatment and psychosocial management/supportive care of ALHIV. With their expressed written consent all these HCPs were included in the study.

### Data analysis

The in-depth interviews were tape-recorded and transcribed verbatim and data analyzed thematically using NVivo version 11. The initial analysis was undertaken by two of the researchers who reviewed the data independently, after which consensus was reached regarding descriptive codes and themes that emerged. Themes were then coded line by line in the data according to the major topics in the interview guide. Data was then charted into a framework matrix using Nvivo software, where data interpretation took place.

## Results

### Socio-demographic characteristics of HCPs

Majority of the HCPs were respectively in the early and middle adulthood ages (*n* = 13, 46.4%) and (*n* = 8, 28.6%). The highest proportions were females and nurses/midwives (*n* = 22, 78.6%) and (*n* = 19. 67.9%), respectively. With regards to their working experience in healthcare, the majority (*n* = 16, 57.1%) have had less than ten years working experience with the highest proportion currently working at the public health Unit (*n* = 23, 82.1%). Although the majority said they are HIV counselors (*n* = 23, 82.1%), less than half (*n* = 11, 47.8%) have received formal training in HIV counseling (Table 1).

**Table 1:** Socio-demographic Characteristics of HCP (*N* = 28)

Parameter	Frequency, n (%)
**Age (years)**	
< 30	5 (17.9)
30–40	13 (46.4)
41–50	8 (28.6)
51–60	2 (7.1)
**Gender**	
Male	6 (21.4}
Female	22 (78.6)
**Category of staff**	
Nurse/Midwife	19 (67.9)
Doctor	1 (3.6)
Biomedical scientist	4 (14.3)
Support staff^a^	4 (14.3)
**Years in healthcare**	
< 5	7 (25.0)
5–10	9 (32.1)
10–15	8 (28.6)
15–20	3 (10.7)
> 20	1 (3.6)
**Current Unit in the hospital**	
Public health unit/ART Clinic	18 (64.3)
Laboratory	4 (14.3)
TB UNIT	2 (7.1)
Wards	4 (14.3)
**Years at the current unit in the hospital**	
< 5	13 (46.4)
5–10	12 (42.9)
10–15	2 (7.1)
15–20	0 (0.0)
> 20	1 (3.6)
**Are you a trained HIV counselor?**	
Yes	23 (82.1)
No	5 (17.9)
**How/Where did you receive training in HIV counseling? (*N*** **= 23)**	
On the job by my colleagues	8 (34.8)
By the National AIDS Control Programme	11 (47.8)
As part of my professional training program	4 (17.4)

^a^These are mainly social workers and peer educators

### Knowledge of HCPs on general adolescent health, education and counseling

Although the majority of the HCPs claim to be knowledgeable about adolescent health issues (*n* = 21, 75.0%), only about a third (*n* = 10, 35.7%) could correctly define who an adolescent is. Only a tenth (*n* = 3, 10.7%) were aware of the widely used adolescent health assessment tool. The majority (*n* = 18, 64.3%) have not received any training on how to work with the adolescent client. Majority (*n* = 26, 92.8%) and (*n* = 24, 85.7%) were respectively, knowledgeable about adolescents’ right to privacy and informed consent (Table 2).

**Table 2:** Knowledge of HCPs on general adolescent health, education and counseling (*n* = 28)

Parameter	Frequency, n (%)
**Do you consider yourself knowledgeable about adolescent health issues?**	
Yes	21 (75.0)
No	7 (25.0)
**What is the correct definition for adolescents?**	
Persons aged 10–19 years	10 (35.7)
Persons aged 13–19 years	18 (64.3)
**Have you received any formal training on adolescent health?**	
Yes	14 (50.0)
No	14 (50.0)
**Have you received any training on how to work with the adolescent client?**	
Yes	10 (35.7)
No	18 (64.3)
**Does the adolescent have the right to privacy?**	
Yes	26 (92.8)
No	1 (3.6)
I do not know	1 (3.6)
**Does the adolescent have the right to informed consent?**	
Yes	24 (85.7)
No	2 (7.1)
I do not know	2 (7.1)
**Are all adolescents the same in terms of characteristics and behavior?**	
Yes	5 (17.9)
No	20 (71.4)
I do not know	3 (10.7)
**Are you aware of any Laws and Policies related to Adolescents in Ghana?**	
Yes	19 (67.9)
No	5 (17.9)
I do not know	4 (14.2)
**Are counseling approaches/techniques used in the general adolescent health the same as those used for ALHIV?**	
Yes	11 (39.3)
No	12 (42.8)
I do not know	5 (17.9)
**Which of these is an adolescent health assessment tool?**	
WHODAS	1 (3.6)
HEADSSS	3 (10.7)
WHOQAS	0 (0.0)
ADOLETS	2 (7.1)
No idea	22 (78.6)

**Fig. 2:**
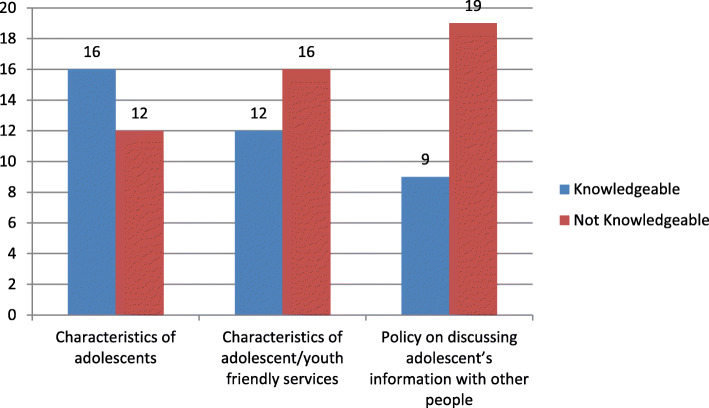
Knowledge level of HCWs obtained from scoring of multiple response type questions (*N* = 28)

Figure 2 further revealed that although majority (*n* = 16, 57.1%) were knowledgeable about the characteristics of adolescents in terms of growth and development and general behavior, majority (*n* = 16, 57.1%) lacked knowledge on the characteristics of adolescent or youth-friendly services and more than two thirds (*n* = 19, 67.8%) did not know about any state law or policy related to discussing Adolescents’ health/care information with third parties (Fig. 2).

### Post training knowledge

During the post-training interviews, participants reported an improved understanding of the characteristics of an adolescent-friendly site and basic principles for ALHIV care.

*“It [*adolescent friendly services] *must be accessible to all*, *in terms of location. It should be situated in a place that is not too open or too far from access to transport. There should be directional signs to guide. It should not be expensive too. Services provided must be appropriate to the developmental and health needs of the adolescent. Services must be acceptable to the adolescent.” (P010, Male HTC Counselor).*

Participants were also able to correctly describe the widely used adolescent health assessment tool “the HEEADSSS (a mnemonic for Home, Education/Employment, Activities, Drug use and abuse, Sexuality, Safety, and Suicidality/Signs of depression).”

*"It is a very good tool because it covers all aspects of adolescent life and helps in knowing whether the adolescent is at risk of a particular health problem or not" (P001, HTC/Adherence counselor)*

Also, most of them expressed that they were first introduced to a psychosocial health assessment tool with adolescent-specific focus, during the training. They also found the training on the HEADSSS and its inclusion in the care of ALHIV to be very beneficial:

*"I was first introduced to the tool during the training. It is a good tool and very useful. This is because it will enable us to perform a comprehensive assessment of the adolescent client quickly and to identify any threat to their physical, psychological or social life." (P005, Female HTC/TB Drug Adherence Counselor)*

The perceived improved knowledge and competence in the psychosocial assessment is heightened in participants’ readiness and ability to use the HEADSSS tool for routine assessment of ALHIV, which was very reassuring.

*“I am ready to use the tool but will need the manual or a checklist as a guide.” (P005, Female HTC/TB Drug Adherence Counselor)*

*I have started using the tool. At first, it appears to be lengthy, but with time when one gets used to the set of questions, I believe the time spent will be shortened. A checklist of the abridged version will be very much appreciated." (P007, Female* HTC/ART Adherence Counsellor*)*

### HCPs practices regarding adolescents counseling and other care provision

Regarding the issue of seeking adolescents’ consent during care, only a little over a third of the HCPs (*n* = 10, 35.7%) always sought the adolescents’ consent in all care related activities whilst a little over a quarter (*n* = 8, 28.6%), said they always sought their consent of adolescents if personal health information is to be disclosed outside the health care team. Closely linked, only a few (*n* = 5, 17.9%) always informed adolescents of their diagnosis, the treatment process and prognosis in the presence of their guardians while the majority (11, 39.2%) only do so occasionally (Table 3).

**Table 3:** HCPs Practices Regarding Adolescents Counseling and Other Care Provision (*n* = 28)

Parameter	Frequency, n (%)
**How often do you seek the consent of adolescents in all care related activities?**	
Always	10 (35.7)
Most of the times	4 (14.3)
Sometimes	10 (35.7)
Never	4 (14.3)
**How often do you seek the consent of adolescents if personal health information is to be disclosed outside the health care team?**	
Always	8 (28.6)
Most of the times	4 (14.3)
Sometimes	9 (32.1)
Never	7 (25.0)
**Who have you ever shared an adolescents’ health information with?**	
Other health care team members	10 (35.7)
Parents/guardians	6 (21.4)
School teacher	0 (0.0)
I have never	12 (42.9)
**How often do you discuss ALHIV information with colleague HWCs?**	
Always	1 (3.5)
Most of the times	2 (7.1)
Sometimes	16 (57.1)
Never	9 (32.1)
**Does the adolescent have the right to make healthy choices for him/herself?**	
Yes	24 (85.7)
No	1 (3.5)
I do not know	3 (10.7)
**Will you deny the adolescent of a service that is his/her health choice, if you think such a healthy choice will harm him/her?**	
Yes	17 (60.6)
No	2 (7.1)
Not sure	9 (32.1)
**Do you inform adolescents of their diagnosis, the treatment process, and prognosis in the presence of their guardians?**	
Always	5 (17.9)
Most of the times	6 (21.4)
Sometimes	11 (39.2)
Never	6 (21.4)
**How often do you ask about the psycho-social needs of ALHIV that you see during clinic hours?**	
Always	5 (17.9)
Most of the times	9 (32.1)
Sometimes	9 (32.1)
Rarely	5 (17.9)
**How often do you carry out health education for adolescent clients?**	
First time I meet an adolescent client	1 (3.5)
Every time I meet adolescent clients	10 (35.7)
Occasionally, depending on their needs	10 (35.7)
Occasionally depending on the workload at the clinic	4 (14.3)
Rarely	3 (10.7)
**Does your facility have protocol/guidelines for general adolescent counseling?**	
Yes	13 (46.4)
No	7 (25.0)
I do not know	8 (28.6)
**Are the available counseling guidelines in your facility clear or specific on how to counsel an ALHIV?**	
Yes	4 (14.3)
No	13 (46.4)
I do not know	11 (39.2)

### Post training practices

The post-training interview revealed better-informed participants who could identify that good HCP’s qualities, respect for adolescents’ rights, ensuring adolescent-friendly environment, adherence to adolescent related policies, routine psychosocial assessment and health education as being an integral part of ALHIV care. These are exemplified in the quote;

“*There should be a holistic approach to care and need to factor in their growth and developmental needs. Every encounter with them is an opportunity for health communication. We need to adhere to national adolescent health policy, such as issues of when to maintain confidentiality or the type of care the HCW can give to the adolescent with or without parental consent” (P006, Female HTC/ART Adherence Counsellor/ Adolescence Health Focal Person)*

### Perception/attitude of HCPs towards working with adolescents

Although the majority (*n* = 21, 75.0%), said they had what it takes to work with the adolescents, almost all (*n* = 26, 92.9%), agreed that working with adolescents can be very challenging. The majority (*n* = 21, 75.0%) also agreed that HCPs who have received training in adolescent health counseling are more likely to provide quality care. More than half (*n* = 16, 57.2%) were of the view that for every decision to be taken regarding an adolescent’s health, the parents/guardians must know, so long as he/she is a minor. A higher proportion (*n* = 24, 85.7%) also agreed that a routine administration of a checklist on psychosocial assessment would help track their needs (Table 4).

**Table 4:** Perception/Attitude of HCWs towards working with Adolescents (*n* = 28)

Parameter	Frequency, n (%)
**I have what it takes to work with adolescents**	
Disagree	7 (25.0)
Neutral	0 (00.0)
Strongly Agree	21 (75.0)
**Working with adolescents can be very challenging**	
Disagree	2 (7.1)
Neutral	0 (0.0)
Agree	26 (92.9)
**Working with adolescents can be fun**	
Disagree	6 (21.4)
Neutral	2 (7.1)
Agree	22 (78.5)
**How ready are you to accept calls from your adolescent clients at any time in the day?**	
Not ready	1 (3.5)
A little ready	4 (14.3)
Ready	11 (39.3)
Very ready	12 (42.9)
**How committed are you to adolescent health-related activities**	
Very committed	16 (57.1)
Somehow committed	9 (32.1)
Not committed	3 (10.7)
**How worried are you about adolescent becoming dependent on you because of your caring nature**	
Not worried	6 (21.4)
A little worried	15 (53.6)
Very worried	7 (25.0)
**HCPs who have received training in adolescent health counseling are more likely to provide quality care**	
Disagree	7 (25.0)
Neutral	0 (00.0)
Agree	21 (75.0)
**HCPs who have not received training in adolescent health counseling are more likely to have difficulties working with adolescents**	
Disagree	5 (17.9)
Neutral	2 (7.1)
Agree	21 (75.0)
**For every decision to be taken regarding an adolescent’s health, the parents/guardians must know, so long as he/she is a minor**	
Disagree	8 (28.6)
Neutral	4 (14.3)
Agree	16 (57.2)
**Because of the workload at the clinic, it is very difficult to have one on one quality time engaging adolescent clients in discussing their health**	
Disagree	4 (14.3)
Neutral	4 (14.3)
Agree	20 (71.4)
**Because of their peculiar issues/challenges, it is always better to have a separate healthcare facility/unit for only adolescents**	
Disagree	3 (10.7)
Neutral	4 (14.3)
Agree	21 (75.0)
**A checklist on psychosocial assessment to be administered routinely would help track their needs**	
Disagree	2 (7.1)
Neutral	2 (7.1)
Agree	24 (85.7)
**Specific HCPs should be assigned only to attend to the needs of ALHIV at the facility to facilitate quality care**	
Disagree	6 (21.4)
Neutral	0 (00.0)
Strongly Agree	22 (78.6)

### Post-training perception/attitude

In the post-training interview, participants were asked to compare their pre and post-training perception and experiences with regards to ALHIV care, which revealed HCPs perception on increased practice related confidence levels and readiness to implement new knowledge and skills gained. This is evident in the following excerpts:

*"I can now confidently work at the ALHIV clinic. Because I have gained new knowledge on how to probe for more information that will form the basis to provide needed and quality care to them. I am ready to put to practice all that I have learned. However, it will be better if we are provided with a manual to guide us." (P009, Female HTC/ART Adherence Counsellor)*

“*Yes, I now have what it takes to care for my ALHIV clients. This is because I can now apply the concepts and strategies taught and also use the new assessment tool as a guide when dealing with them.” (P008, Female Dietician*/*Diet therapy Counsellor)*

*“Yes, to a greater extent. I can communicate with them better. I will also apply the various strategies and skills when caring for them. I can also better assess my adolescent client with the HEADSSS tool. I have had a better understanding of what the adolescent health policy says especially about adolescent confidentiality and decisions they are entitled, so I now know where to draw the line.” (P001, Male HTC/Adherence Counselor).*

Facility-specific gaps and the related potential threat to ALHIV retention into care or poor care outcome was also revealed. In their recommendations, all participants interviewed used knowledge gained to advocate for a friendly and well-resourced ART site with adolescent-specific focused care for the ALHIV client. The interplay between availability of job aids, working guidelines/manuals, adolescent-friendly environment and quality supportive care to the ALHIV clients was made evident in the following comment:

“*Our facility lack manuals for ALHIV care to guide HCWs, as well as educational materials and other recreational activities for ALHIV especially during waiting. We have serious issues with space. Our setting is not helping us to provide a more comprehensive and quality care to our clients. We, therefore, need help from NACP and other stakeholders, for a friendlier adolescent site to support our adolescent have a better life.” (P001, Male HTC/Adherence Counselor)*

## Discussion

This study has highlighted the importance of continuous professional education of healthcare workers involved in the care of clients with complex needs. The study found reported improvement in key aspects of HCPs knowledge, attitudes, and experiences, post-training. HCPs recognized the relevance of implementing evidence-based strategies in ALHIV care.

In our study, and in particular before the training, we observed limited knowledge and skills in specific aspects of ALHIV care and training needs in a variety of adolescent health issues. Although the majority of the HCPs claimed to be knowledgeable about adolescent health issues and the characteristics of adolescents in terms of growth, development and general behavior, perceived knowledge was generally deficient in areas such as the characteristics of adolescent/youth-friendly services, state laws or policies related to adolescents’ health or care. Also, the majority were unaware that the basic counseling approaches/techniques used in general adolescent health also apply to ALHIV. This deficit could be attributed to a lack of relevant training and preparation [7﻿, 13﻿].

It is therefore unsurprising that, in the post-training interviews, interviewees strongly recommended the availability of job aids and ALHIV- specific guidelines and protocols. It was also observed that some of the practices of HCPs in their care of ALHIV were sub-optimal. For instance, only a few always sought consent from the adolescent in all care-related activities or scenarios when potentially, personal health information was to be disclosed to a third party outside the immediate healthcare team. It was also noted that those who routinely assessed the psycho-social needs of their ALHIV clients during clinic hours were in the minority, and only about a third routinely carried out some form of health education for their adolescent clients during clinic hours. However, the post-training interview revealed participants referring to these as integral parts of ALHIV care. Our findings, therefore, have long-term implications for ALHIV care provision in Ghana. Understanding the basic principles and approaches to adolescent-specific care interventions will enable HCPs to be successful in supporting these clients and tailor care to increase adherence to treatment, improve psychosocial care, and improve the quality of life of ALHIV [6].

Lack of training could also explain why only a small proportion (25%) expressed confidence in communicating or working with the adolescents. In this study, articipants were not confident at assessing, communicating and working with adolescents before the training. This perceived lack of confidence could likely be linked to low knowledge levels or lack of training regarding the care of the adolescent client. The direct linkage of low confidence/competence levels of HCPs in caring for adolescents to low knowledge levels and inadequate preparation has been reported elsewhere in the literature, suggesting that insufficient experience and training in adolescent-specific care amounts to sub-optimal management of adolescent health problems [5, 6]. Addressing this gap in our intervention resulted in perceived improvement in confidence levels post-training. The capacity-building of the HCPs on adolescent processes and practical usage of assessment tools reflected in positive outcomes as reported in the post-training interviews.

Worthy of note are participants who reported improved knowledge of selected areas covered in the study, including the HEEADSSS psychosocial health assessment approach, characteristics of adolescent-friendly sites, national policies regarding adolescent health, and general approach to ALHIV care. Before the training, only a few knew about the HEEADSSS and for all those interviewed, their first time of knowing about the HEEADSSS was during the training. Given the vulnerabilities and unique needs of ALHIV, HCPs lack of awareness of basic available adolescent-based tools to facilitate routine assessment is a crucial and urgent dearth to be addressed in any healthcare setting that delivers ALHIV care. According to Sacks and Westwood [14], the HEEADSSS assessment tool, which is one such tool, has been validated as effective in promoting health workers to probe aspects of an adolescent’s psychosocial life which they might otherwise forget or ignore. This tool has great potential to help HCPs identify existing and potential problems in our ALHIV population and to suggest modifications to be made to their care approach. Timely and swift interventions for HCPs can increase awareness and usage of these essential tools, as shown in our study, where post-training, all participants now demonstrated familiarity with the assessment tool and expressed their readiness in its implementation at the workplace.

With regards to the challenges the HCPs encountered in the course of providing care to the ALHIV clients, four problematic situations were identified: problems of communication with the ALHIV client, treatment adherence-related issues, physical care environment, and unavailability of job aids and written protocols to facilitate their work. Some experiences highlighted by HCPs included; unease and lack of adequate knowledge and skill in communicating sensitive issues (such as sex, HIV status disclosure), and ways to optimize adherence to treatment. While the latter two problematic situations originate from facility-based deficiencies, addressing them would help in solving the problems in dealing with the ALHIV client. The discomfort many healthcare providers have in discussing sexual and reproductive health with the adolescent clients, particularly in some African settings has been reported in the scientific literature [15–17].

HCPs challenges encountered in the course of delivering health care for young people with HIV including ALHIV have been widely identified [5–7]. The WHO has reported that HCPs including those experienced in caring for adults with HIV, are often ill-equipped to support the healthcare needs of adolescents [6, 7]. Poor prioritization of adolescents in national policies for scaling-up HIV testing and treatment services compounds the problem.

During the post-training evaluation, participants felt they have what it takes to address the ALHIV care specific challenges and were very willing to apply the adolescent-specific strategies and assessment tools in their day to day practices. In “Knowles theory of adult learning,” Knowles and colleagues, refers to the importance of motivation and the readiness to learn [18]. Our study highlights that HCPs are motivated and very valuable when it comes to the establishment of ALHIV chain of survival, which needs to be encouraged. In a survey of 10 countries in Sub-Saharan Africa on mapping HIV services and policies for adolescents, the authors reported on the training needs of HCPs involved in ALHIV care and how it will enhance the provision of quality care services to these clients [19]. Future studies could be to investigate using a prospective design, the impact of such training of HCP on the care ALHIV receive from the perspective of the adolescents themselves which was not done in this study due to limitations of time and resources.

### Limitations

A significant strength of this study was its census approach which enabled views of all members of the population of interest to be included. It is, however, acknowledged that this is a small study which did not make use of a formal pre and posttest approach. In addition, the views of ALHIVs were not incorporated in the design of the content of the training which would have possible further improve the potential impact.

## Conclusions

This study has demonstrated that interventions that maximize adolescent-specific approaches/strategies for HCPs as part of routine care are effective in increasing their knowledge, skill, and confidence. Availability and training in usage of ALHIV healthcare algorithms complemented this gain and reduced the gaps identified at the workplace. Healthcare managers and relevant stakeholders could adopt this strategy on a national basis, and these can be replicated in similar health care settings in SSA in a bid for the continent to accelerate attainment of the sustainable development goals.

## Data Availability

The datasets used and analyzed during the current study are available from the corresponding author on reasonable request.

## Abbreviations

ALHIV: Adolescents living with HIV
ART: Antiretroviral Therapy
CCTH: Cape Coast Teaching Hospital
GHS: Ghana Health Service
HCPs: Healthcare Providers
HIV/AIDS: Human Immunodeficiency Virus/Acquired Immune Deficiency Syndrome
HTC: HIV testing and Counselling
NACP: National AIDS/STI Control Program
PMTCT: Prevention of Mother to Child Transmission of HIV
SSA: Sub-Saharan African
TB: Tuberculosis
